# Google Trends on Obesity, Smoking and Alcoholism: Global and Country-Specific Interest

**DOI:** 10.3390/healthcare9020190

**Published:** 2021-02-09

**Authors:** Fabio Fabbian, Pedro Manuel Rodríguez-Muñoz, Juan de la Cruz López-Carrasco, Rosaria Cappadona, María Aurora Rodríguez-Borrego, Pablo Jesús López-Soto

**Affiliations:** 1 Department of Medical Sciences, University of Ferrara, 44121 Ferrara, Italy; rosaria.cappadona@unife.it; 2 Department of Nursing, Instituto Maimónides de Investigación Biomédica de Córdoba, 14005 Córdoba, Spain; z52romup@uco.es (P.M.R.-M.); juan.delacruz@imibic.org (J.d.l.C.L.-C.); en1robom@uco.es (M.A.R.-B.); 3 Clinica Medica Unit, Department of Medicine, Azienda Ospedaliero-Universitaria “S. Anna”, 44121 Ferrara, Italy; 4 Nursing Area, Faculty of Health Sciences, Universidad Pontificia de Salamanca, 37002 Salamanca, Spain; 5 Department of Nursing, Universidad de Córdoba, 14004 Córdoba, Spain; 6 Hospital Universitario Reina Sofía de Córdoba, 14004 Córdoba, Spain

**Keywords:** obesity, diet, alcoholism, smoking, lifestyle behavior, Google Trends

## Abstract

Unhealthy habits or lifestyles, such as obesity, smoking, and alcohol consumption, are involved in the development of non-communicable diseases. The aim of this study was to analyze different communities’ interest in seeking obesity, smoking, and alcohol-related terms through relative search volumes (RSVs) of Google Trends (GT). Internet search query data on obesity, smoking, and alcohol-related terms were obtained from GT from the period between 2010 and 2020. Comparisons and correlations between different topics were calculated considering both global searches and English-, Spanish-, and Italian-speaking areas. Globally, the RSVs for obesity and alcohol-related terms were similar (mean RSVs: 76% and 77%), but they were lower for smoking (65%). High RSVs were found in winter for obesity and smoking-related terms. Worldwide, a negative correlation was found between alcohol and smoking terms (r = −0.72, *p* < 0.01). In Italy, the correlation was positive (r = 0.58). The correlation between obesity and alcohol was positive in all the cases considered. The interest of global citizens in obesity, smoking, and alcohol was high. The RSVs for obesity were globally higher and correlated with alcohol. Alcohol and smoking terms were related depending on the area considered.

## 1. Introduction

Health, according to the World Health Organization [1], is defined as “a state of complete physical, mental, and social well-being, and not just the absence of conditions or diseases”. Lifestyle is a major determinant of health, and integrates healthy and unhealthy habits and behaviors [2]; in fact, the lack of a healthy lifestyle predisposes individuals to public health problems [3].

The biggest contributor to non-communicable diseases globally is unhealthy lifestyle habits, causing approximately 63% of all deaths in the world [4]. The economic impact of non-communicable diseases on health systems has been increasing; therefore, it is important to emphasize that early interventions for risk reduction are necessary, thus “enabling people to increase control over and to improve, their health” (Ottawa Charter for Health Promotion) [5].

Healthy lifestyles should take into consideration a set of factors, such as diet, physical exercise, and toxic habits; the last of which includes use and abuse of alcohol and tobacco [2].

Changes in human lifestyle could be considered one of the main causes of obesity. The prevalence of obesity has increased around the world over the last 50 years, and has raised the risk of diseases such as type 2 diabetes mellitus and hypertension. Lifestyle interventions are necessary to reduce the worldwide burden of obesity. This is mainly based on reducing caloric intake and increasing energy expenditure with physical activity [6,7].

Moreover, death could be associated with toxic habits, and 3 million deaths every year are due to the harmful use of alcohol, resulting in significant social and economic losses for society [8]. Smoking is a different unhealthy habit, and all forms of tobacco consumption are harmful, with tobacco consumption being one of the major causes of disease. Tobacco kills more than 8 million people each year, and approximately 1.2 million non-smokers are exposed to second-hand smoke [9].

The internet is increasingly used among the population to access health-related information, and is a very important source of knowledge for those who seek changes in their lifestyles [10,11], and to search for information about specific conditions [12]. Health professionals must be prepared to consider this instrument when seeking ways to help the population increase their awareness and to critically evaluate the information reported [11].

Infodemiology is a research area focusing on internet users looking for health-related content [13]. Infodemiology uses non-clinical databases such as web searches to allow health scientists to examine a population’s interest in multiple health issues through the internet, especially Google platforms. Evaluation of data obtained from Google has proven to be a useful proxy for lifestyle or behavior, especially in areas difficult to measure with surveys [10,14]. Google Trends (GT) is a popular means for searching data and could be used to examine public interest in multiple health topics [3,10,11,15].

The aim of this study was to analyze the web search trends related to community interest in obesity, smoking, and alcohol worldwide and in English-, Spanish-, and Italian-speaking areas during the period from 2010–2020.

## 2. Materials and Methods

Using GT, we aimed to conduct a descriptive analysis of the global community’s interest in unhealthy lifestyles. To detect such an interest, we compared worldwide relative search volumes (RSVs) obtained using obesity-, alcohol-, and smoking-related search terms.

GT is a free, publicly available internet-based application allowing searches of global or local interest in targeted search terms. Values representing RSVs are obtained, and range between 0 and 100. When an RSV is equal to 0, it means that internet users are not searching significantly for the selected term; on the other hand, an RSV equal to 50 means that half as many searches are carried out for the selected term, compared to the highest volume of searches, represented by an RSV equal to 100. Temporal limitations in searching can be established, and the period of time can vary from weeks to months or years. Different given search terms can be compared by evaluating RSVs. Searching can be associated with different areas and then compared. Data are adjusted for population size; therefore, comparisons between more or less populated areas can be made. To compare relative frequencies and to standardize data between different countries, each data-point is divided by the total searches for a given geographical area and the established interval of time.

We explored GT data using specific language search terms for both global searches and English-, Spanish-, and Italian-speaking areas (Table 1). Search terms were arbitrarily selected by the authors to capture scientific words that define an unhealthy condition but that are also considered popular, i.e., easily understood by the general population. Four terms were selected in the three languages (i.e., English, Spanish, and Italian). Although other terms may be considered common, to avoid heterogeneity, the authors decided to use the corresponding terms in the three languages. The countries analyzed were the United States of America, the United Kingdom, Italy, and Spain. However, Australia and Mexico were also included in the analysis (Figure S1).

For general searches, only 7 terms (3 in English, 2 in Spanish, and 2 in Italian) were used because GT only allows that number. The selection of the keywords was as follows:

− Alcohol keywords: “Alcohol”, “beer”, “drinking”, “cerveza”, “botellón”, “alcol”, and “birra”.
− Smoking keywords: “Smoking”, “tobacco”, “cigarette”, “fumar”, “cigarro”, “fumo”, and “tabacco”
− Obesity keywords: “Obesity”, “fast food”, “calories”, “obesidad”, “calorías”, “sedentarismo”, and “calorie”.

Searching was limited to the category “Health” to avoid non-health-related queries and confounding results. We decided to analyze GT from 19 September 2010 to 20 September 2020, and considering global and specific world areas.

Data analysis was directed to show RSV variations during the study period for comparing different world areas and assessing correlations between RSVs related to obesity, alcohol, and smoking. We correlated the data obtained from GT with those provided by the World Health Organization (WHO), with the intention of comparing the community’s interest in unhealthy lifestyles with officially calculated prevalence data [16,17,18].

The results were plotted in graphs, and the mean and standard deviation (SD) of RSVs were determined. Specifically, RSVs were also evaluated during summer (third week of June to third week of September) and winter (third week of December to third week of March) periods. Significant differences were determined via the Mann–Whitney test. Associations were analyzed by calculation of Pearson correlation or Spearman’s rank correlation coefficients, depending on linearity. All analyses were performed using the free software, R (version 3.5.0, R Foundation for Statistical Computing, Vienna, Austria). A two-tailed threshold of *p* < 0.05 was considered statistically significant.

All the data are freely available online, so no specific permits were required from ethics committees, although in the development of the study, all procedures were carried out with appropriate scientific rigor.

## 3. Results

**Figure 1:**
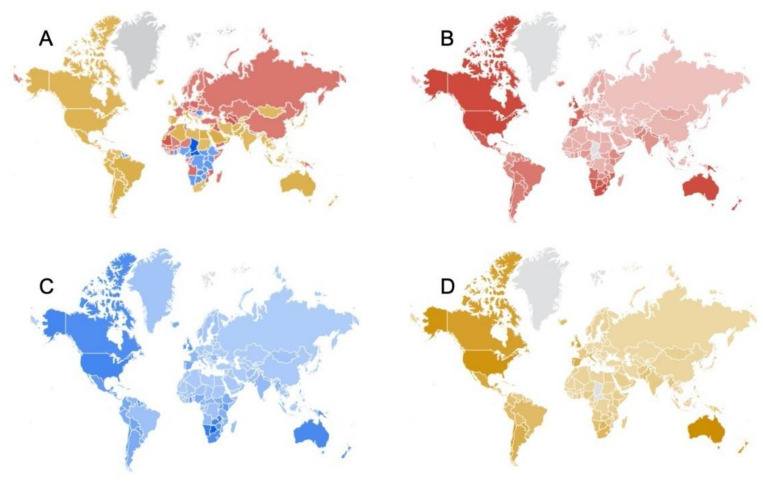
World distribution of relative search volumes (RSVs) from 2010–2020, and related to: (**A**) all terms (obesity, alcohol, and smoking); (**B**) smoking terms (red); (**C**) alcohol terms (blue); and (**D**) obesity terms (yellow). Greater shading indicates RSVs (the figure was captured from Google Trends; Data source: Google Trends (https://www.google.com/trends, accessed on 20 September 2020).

**Figure 2:**
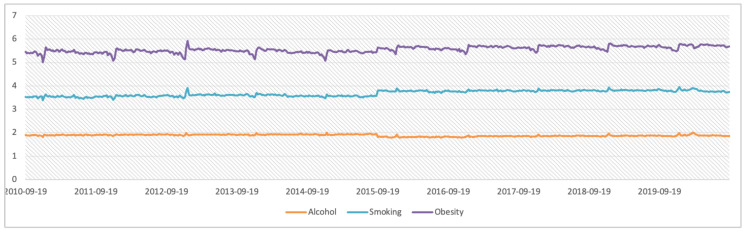
Time series of Google search queries. Search fractions are rescaled between 0 and 100 by the internal processes of Google Trends upon download, although a logarithmic scale was used to represent the results. Note: Google Trends had two methodological improvements (namely, on 1 January 2011, improvement in geographic allocation, and on 1 January 2016, improvement in data collection). Data source: Google Trends (https://www.google.com/trends, accessed on 20 September 2020). The chart legend indicates the colors for each topic.

The mean values and standard deviations of the RSVs for obesity, alcohol, and smoking during the study period in all countries were 76.03 ± 11.17%, 77.38 ± 7.09%, and 65.82 ± 21.30%, respectively. A comparison of the RSVs for obesity, alcohol, and smoking in all countries is shown in Figure 1. Obesity-related terms were the most frequently searched worldwide, and the temporal pattern analysis of the logarithm of RSVs during the analyzed decade is reported in Figure 2, where some interesting variations are shown. Significant differences were found between the RSVs in the summer and winter months, and values were higher during winter for obesity-(79.70 ± 12.79% vs. 77.04 ± 6.80%) and smoke-related terms (70.65 ± 21.64% vs. 63.27 ± 21.05%). No differences were found for alcohol terms.

The correlation coefficient between alcohol and smoking RSVs was negative and highly significant. In the same way, the correlation between obesity and smoking RSVs was negative, although this relationship was weak. Moreover, a weak positive correlation between obesity and alcohol RSVs was found (Table 2).

Correlations between official data from the Global Health Observatory of the WHO and Google Trends are reported in the supplementary Figure S1. The correlation was positive and strong for obesity in Italy, Spain, and the United Kingdom, while for smoking, it was negative in Italy and Spain. In the United States of America, the correlation was positive and strong for alcohol, while it was negative in the United Kingdom (Table 3).

In the case of obesity, we compared the existing annual data from 2004 to 2016 (representing the latest data provided by WHO) [16]. In the case of smoking, we compared specific WHO annual periods (2007, 2010, 2012, 2014, 2016, and 2018) [17]. In the case of alcohol use and abuse, we considered the existing annual data from 2004 to 2018 (representing the latest data provided by WHO) [18]. GT showed RSV values from 2004 onwards (considering that there were measurement changes in 2011 and 2016, and that only the English language was used). It should be taken into account that the GT data included all types of information related to smoking and alcohol use and abuse (both prevention and consumption), while the WHO data recorded only tobacco consumption and alcohol use and abuse [17,18].

**Figure 3:**
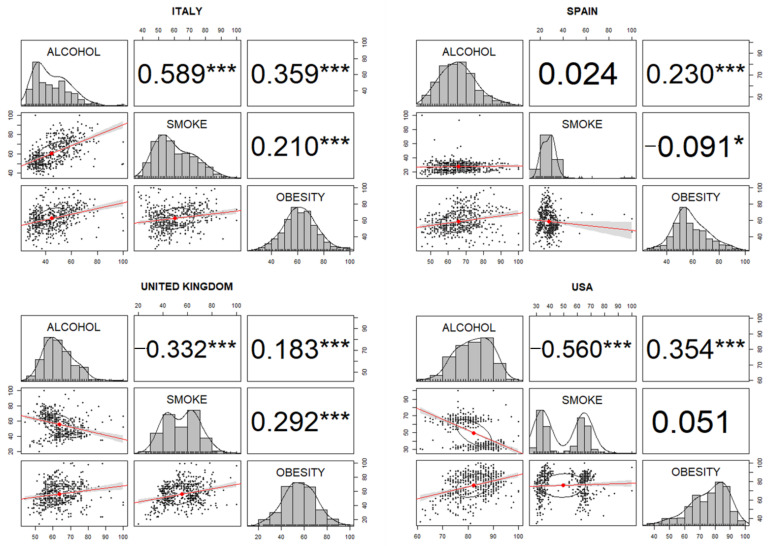
Correlation plots of searches performed by country using country-specific languages. * *p* < 0.05; *** *p* < 0.001.

Finally, we evaluated the results related to English-, Spanish-, and Italian-speaking areas (Figure 3). We found a modest positive significant correlation between alcohol and obesity in all countries studied (United States of America, United Kingdom, Spain, and Italy), and the coefficients were higher in Italy (r = 0.359) and in the United States of America (r = 0.359). Positive correlations were also detected between obesity and smoking terms in the United Kingdom (r = 0.292) and in Italy (r = 0.210). Curiously, the correlation between alcohol and smoking was negative in the United Kingdom (r = −0.332) and the USA (r = −0.560), but was positive in Italy (r = 0. 589).

Global searches using terms in the English, Spanish, and Italian languages showed correlations between alcohol and obesity in all cases (Table 4); being negative in Italian (r = −0.452) and positive in Spanish and English (r = 0.421 and r = 0.254, respectively). Negative correlations between alcohol and smoking were also found in the Spanish (r = −0.585) and English (r = −0.696) languages. We detected weak negative correlations in the global searches between RSVs related to smoking and obesity in Spain (r = −0.193) and Italy (r = −0.157). The same analysis was carried out in Australia (an English-speaking country) and Mexico (a Spanish-speaking country), and we found that Australian search activity was similar to Spanish search activity, while Mexican search activity was similar to Italian search activity (Figure S1).

## 4. Discussion

In this study, we detected that global interest in information about obesity, alcohol, and smoking in the field of health could be considered significant. The latter results should be interpreted considering that the majority of wealthy countries are located north of the equator, and that countries such as Australia behave in the same way as the United States of America or Western countries. Sociodemographic determinants such as education, occupational status, and income have been reported to be related to the prevalence of obesity and unhealthy behaviors, such as alcohol and tobacco consumption [19]. GT evaluates public interest in obesity, smoking, and alcoholism, but such activity cannot be considered equivalent to a traditional epidemiological perspective. Looking for a comparison to validate, we correlated GT results calculated for the United States of America, the United Kingdom, Spain, and Italy with the Global Health Observatory of WHO, and in some cases, we found a significant relationship, suggesting that GT could in some way capture official epidemiological data.

Our results showed variations in the communities’ interests in searching for terms related to obesity, smoking, and alcohol, but explaining these findings is difficult. There are several variables to consider, from interest to electronic cigarettes to changes in GT’s manner of operation. RSVs deal with searching information at the population level, and they are influenced by media coverage. It has been reported that benefits are overestimated, claims are exaggerated, risk is underestimated, and conflicts of interest are not declared [20].

During the last decade, interest has not changed, and interest in smoking and alcohol was distributed in an opposite way. The latter relationship was confirmed in the United States of America; however, even though Italy and Spain are two similar countries in southern Europe, the interests of the two populations were different. Italians seemed to search for both alcohol and smoking information, while Spanish people did not.

In the global community, the most-searched terms were related to obesity. These terms encompass the major health problem of obesity and overweightness, defined as “abnormal or excessive accumulation of fat that can be harmful to health”, and are due to an energy imbalance between consumed and expended calories [21].

The prevalence of obesity worldwide, which has tripled worldwide since 1975, could be the force driving the global community’s search for information.

The RSVs related to obesity had higher values in American and Mediterranean countries. According to several authors [22,23], the prevalence of obesity and overweightness in the North American area is high. However, our data seemed to suggest that people from that area were aware of the problem, as their RSVs were high. On the other hand, even though, in the Mediterranean area, diet could be considered healthy [24], the prevalence of obesity in children is high due to the high intake of free fats, sugar, and processed meats [25,26]. Looking for a remedy for such a problem could justify the recorded RSVs.

During the last decade, there was an increase in searches for all terms related to “health” in the first months of each year; however, there were differences between the searches carried out in winter and summer, with such differences being significantly greater for smoking and obesity. Such periods may be subsequent to vacations and holiday times, during which it is possible to detect weight gain [27].

We must underscore the lack of information available to the worldwide population. Although alcoholic beverages have a high energy content relative to their calories [28,29], and although alcohol use could be a risk factor for obesity [30], we found a weak correlation between alcohol and obesity.

Analyzing the term “smoking”, we could not see a wide variation of interest during the period of the study, but a small peak in RSVs was recorded at the beginning of 2013. This peak could be due to the anti-smoking policies that were implemented that year in Russia [31], Chile, and Uruguay [32]. Several authors stated that in countries where anti-smoking control policies were implemented, smoking prevalence decreased [33,34,35]. In 2016, Troelstra et al. [36] found a significant increase in RSVs after the introduction of anti-smoking control policies, suggesting that these measures had an effect on the rate of search for information. However, the relationship between information and smoking cessation rates is still a matter of debate [37]. Finally, it should be taken into account that electronic cigarettes have become popular. It was reported that new communication means could be crucial for improving knowledge about this unhealthy habit [38].

Globally, RSVs related to alcohol and smoking were negatively correlated, except in Italy. This negative relationship between these two dangerous habits was not in agreement with studies reporting a strong relationship between them due to social context, especially during the time of youth, when poly-consumption of alcohol and tobacco is frequent [39,40]. Smoking is a risk factor for alcoholism, and alcohol consumption is a risk factor for becoming a smoker [41,42]. Alcohol increases the rate at which the body breaks down nicotine, causing it to remain active in the body for a shorter time, thereby stimulating tobacco use [39]. On the other hand, environmental factors such as economic development, cultural and social aspects including religious beliefs, availability of alcohol and the effectiveness of alcohol policies impact smoking and alcohol use and abuse [43]. In the case of alcohol consumption, RSVs were high for sub-Saharan African countries (Southern and Western African countries); results that were in agreement with Shield et al.’s [43] findings of high alcohol intake in populations from these regions and Eastern European countries. RSVs associated with smoking showed that Asian and Central and Northern European countries had high values, in agreement with a recent survey [44] that suggested that European and Southeast Asian regions, such as China, India, and Indonesia, have a smoking prevalence significantly higher than the mean worldwide frequency.

In this study, we have to take into account several limitations. First, we tested public global interest describing GT data using English, Spanish, and Italian search terms and merely reported RSVs; therefore, it was not possible to detect any cause–effect relationship. Second, although our study investigated across an entire decade, any interpretation of our population of interest could not be based on definitive profiles of the different people performing the searches. GT does not record information about the identity, internet protocol address, or specific physical location of any user. Third, we limited our research to three different languages, and only seven terms could be included in the global search, making it difficult to generalize our results. Fourth, we did not consider that different areas tend to drink alcohol in different ways, and we did not compare RSVs related to beer, wine, or hard alcohol in the different geographical regions. Fifth, we selected four terms in three different languages, English, Spanish, and Italian, but we are conscious that other terms may be considered common. However, in order to avoid heterogeneity, we decided to use the corresponding words in the three languages. Search terms were arbitrarily selected by authors as scientific words defining an unhealthy condition, but also considered popular, i.e., easily understood by the general population. On the other hand, searching of non-specific keywords and those mostly attributable to informative purposes, could lead to non-specific results and methodological problems. Big data analytics is beyond our competence, but integrating information of the top online health search requests from different countries, languages, and time periods, might in our opinion shed light on the dynamics of a community’s knowledge about healthy lifestyle. Finally, the technology used in this study is Web 1.0, and analysis of Web 2.0 technology, such as Twitter or other social media, could result in different findings.

## 5. Conclusions

Based on the objectives proposed in this study, it can be concluded that the global community’s interest in obesity, smoking, and alcoholism is significant, suggesting a consciousness about these dangerous habits. Of the three problems observed in this study, the level of interest in obesity was highest, offering a clear reflection of the increase in this health problem globally.

Unexpectedly, searches for smoking were negatively correlated with alcohol; however, this was not true in the case of Italy.

Interestingly, the number of searches varied in intensity depending on the period of the year, the season, and the tightening of regulations governing consumption.

In terms of its implications for health practice, infodemiology could be a useful instrument for investigating populations’ interest in a specific topic. Such information could help healthcare professionals establish proposals for disease prevention and health promotion, and health care authorities could consider GT for national and subnational trend analysis using the local language for early interception of a population’s of interest with respect to unhealthy habits.

## Figures and Tables

**Table 1:** Specific language terms used for country searches.

Categories	English Terms	Spanish Terms	Italian Terms
Alcohol keywords	Alcohol, beer, drinking, binge drinking	Alcohol, cerveza, borracho, botellón	Alcol, birra, ubriaco, binge drinking
Smoking keywords	Smoking, tobacco, cigarette, vaping	Fumar, tabaco, cigarro, vapeo	Fumo, tabacco, sigaretta, svapo
Obesity keywords	Obesity, fast food, calories, sedentarism	Obesidad, comida basura, calorías, sedentarismo	Obesità, fast food, calorie, sedentarietà

**Table 2:** Correlation coefficients between the relative search volumes (RSVs) related to the obesity-, alcohol-, and smoking-related search terms registered during the study period.

Categories	RSVs for Alcohol	RSVs for Smoking	RSVs for Obesity
RSVs for alcohol	1	−0.728 *	0.327 *
RSVs for smoking	−0.728 *	1	−0.217 *
RSVs for obesity	0.327 *	−0.217 *	1

* *p* < 0.01.

**Table 3:** Correlations between official data (The Global Health Observatory–World Health Organization) and Google Trends.

Obesity	Italy–GT	Spain–GT	UK–GT	USA–GT
Italy–WHO	0.848 **			
Spain–WHO		0.659 *		
UK–WHO			0.824 **	
USA–WHO				0.044
**Smoking**				
Italy–WHO	−0.829 *			
Spain–WHO		−0.943 **		
UK–WHO			0.371	
USA–WHO				0.486
**Alcohol**				
Italy–WHO	−0.066			
Spain–WHO		−0.269		
UK–WHO			−0.622 *	
USA–WHO				0.748 **

Note: WHO: World Health Organization; GT: Google Trends; UK: United Kingdom; USA: United States of America. Spearman’s rank correlation coefficient was used. * *p* < 0.05; ** *p* > 0.01.

**Table 4:** Correlation coefficients of searches performed using country-specific languages (English, Spanish, and Italian).

ENGLISH	RSVs for Alcohol	RSVs for Smoking	RSVs for Obesity
RSVs for alcohol	1	−0.696 *	0.254 *
RSVs for smoking	−0.696 *	1	-0.068
RSVs for obesity	0.254 *	−0.068	1
**SPANISH**			
RSVs for alcohol	1	-0.585 *	0.421 *
RSVs for smoking	−0.585 *	1	−0.193 *
RSVs for obesity	0.421 *	−0.193 *	1
**ITALIAN**			
RSVs for alcohol	1	0.098	−0.452 *
RSVs for smoking	0.098	1	−0.157 *
RSVs for obesity	−0.452 *	−0.157 *	1

* *p* < 0.05.

## Data Availability

The data presented in this study are available on request from the corresponding authors.

## Supplementary Materials

The following are available online at https://www.mdpi.com/2227-9032/9/2/190/s1, Figure S1: Correlation plots of searches performed in Australia and Mexico.
