# Supplementary figures and images for: Google Trends on Obesity, Smoking and Alcoholism: Global and Country-Specific Interest
Source: Healthcare (Basel). 2021 Feb 9;9(2):190. doi: 10.3390/healthcare9020190 (PMC7916197; doi:10.3390/healthcare9020190)

**Figure S1.** Correlation plots of searches performed in Australia and Mexico. \*\*\*p<0.001

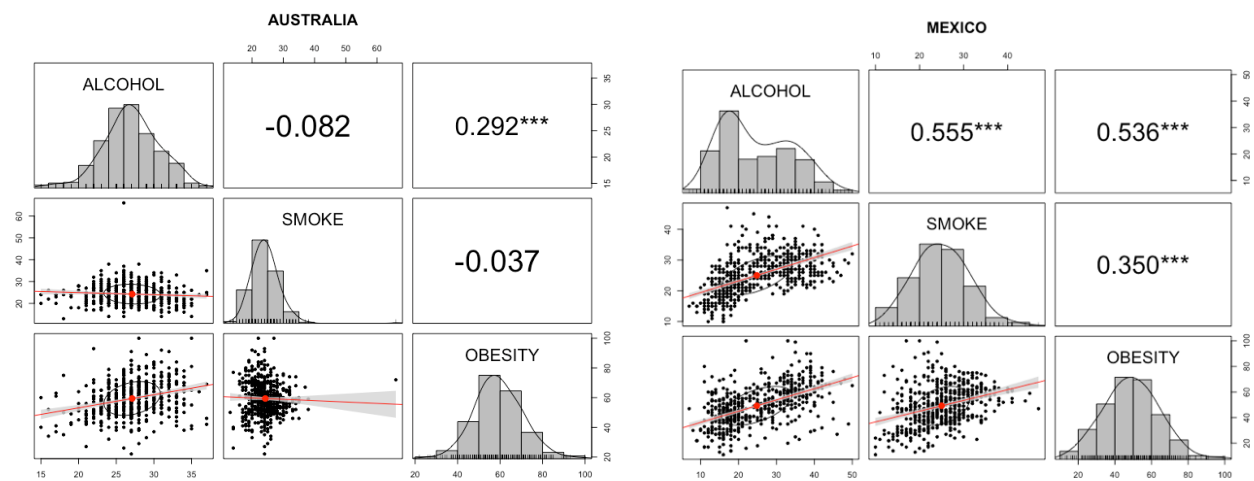

Supplement: Supplementary file 1 [file healthcare-09-00190-s001.pdf]
